# Reference Diameters of the Abdominal Aorta and Iliac Arteries in Different Populations

**DOI:** 10.3390/jcm15020518

**Published:** 2026-01-08

**Authors:** Hyangkyoung Kim, Sungsin Cho, Jin Hyun Joh

**Affiliations:** 1 Department of Surgery, Ewha Womans University College of Medicine, Ewha Womans University Medical Center, Seoul 07985, Republic of Korea; hkkim77@ewha.ac.kr; 2 Department of Surgery, Kyung Hee University Hospital at Gangdong, Seoul 05278, Republic of Korea; 01ssunny@naver.com

**Keywords:** aortic aneurysm, aortic diameter, racial differences, intervention thresholds, aneurysm rupture, population-specific thresholds

## Abstract

Aortic and iliac artery aneurysms are potentially fatal conditions requiring precise timing for intervention. Current guidelines for repair, including those from SVS, ESVS, and ACC/AHA, rely on fixed diameter thresholds primarily derived from Western populations. However, growing evidence shows that both aortic and iliac dimensions vary significantly among racial and ethnic groups. East Asian individuals generally present with smaller baseline vessel diameters and may be at risk of rupture at smaller sizes, while African American and Hispanic populations exhibit distinct remodeling patterns and risk profiles. This narrative review synthesizes the current literature on variations in aortic and iliac artery diameters, aneurysm prevalence, and rupture risk across racial groups. It examines the limitations of universal thresholds for repair, highlights the underrepresentation of non-Caucasian populations in early imaging registries, and introduces alternative, population-specific definitions of aneurysmal disease.

## 1. Introduction

Abdominal aortic aneurysm (AAA) is a common and potentially life-threatening vascular condition characterized by progressive dilation of the abdominal aorta, with rupture representing a catastrophic event associated with mortality rates exceeding 80% [1,2]. AAA remains a major public health concern, particularly in aging populations, where its prevalence increases with age and is strongly associated with smoking, hypertension, and atherosclerosis [3,4]. Population-based screening programs in Western countries have demonstrated that early detection and elective repair significantly reduce aneurysm-related mortality, reinforcing the importance of accurate diagnostic and therapeutic thresholds [5,6].

Current management strategies for AAA rely heavily on fixed diameter criteria to determine the timing of intervention. Elective repair is generally recommended when the aneurysm reaches 5.5 cm in men or demonstrates rapid expansion, based largely on the results of landmark randomized controlled trials such as the UK Small Aneurysm Trial and the Aneurysm Detection and Management trial [7,8]. These thresholds have been incorporated into major clinical guidelines, including those from the Society for Vascular Surgery (SVS), European Society for Vascular Surgery (ESVS), and American College of Cardiology/American Heart Association (ACC/AHA) [6,9,10].

However, it is increasingly recognized that these seminal studies were performed predominantly in Caucasian male populations from Western countries, raising concerns regarding the universal applicability of these thresholds to other racial and ethnic groups. In the era of globalized medicine and increasingly diverse patient populations, it has become clear that vascular anatomy, aneurysm prevalence, growth patterns, and rupture risk may significantly differ across races. Growing evidence suggests that baseline diameters of the abdominal aorta and iliac arteries vary substantially among different populations, even after adjustment for conventional anthropometric factors such as height, body surface area (BSA), and age. In particular, East Asian populations consistently demonstrate smaller native aortic and iliac artery diameters compared with Western cohorts [11,12], while African American and Hispanic populations exhibit distinct vascular remodeling patterns and disease behavior [13,14]. These differences may have important clinical implications when applying uniform size-based criteria for aneurysm diagnosis and repair. A fixed threshold that is safe for one population may represent a disproportionately advanced degree of dilation in another, potentially leading to delayed intervention and increased rupture risk. Conversely, rigid thresholds may lead to unnecessary treatment in individuals with inherently larger baseline vessel dimensions. Importantly, the field has already embraced a precedent for individualized thresholds through the evolution of sex-specific recommendations [6,10]. Current guidelines now recognize that women have a higher risk of aneurysm rupture at smaller diameters and therefore advocate for lower surgical thresholds in selected cases. This shift highlights a broader conceptual change in aneurysm management—moving away from a “one-size-fits-all” paradigm toward a more personalized, anatomy-based approach. Despite this progress, racial and ethnic variability has not yet been systematically incorporated into guideline recommendations.

Furthermore, most early imaging registries and clinical trials that informed current thresholds underrepresented non-Caucasian populations, resulting in limited data on normal aortic dimensions and aneurysm behavior in these groups. This knowledge gap is particularly relevant given the increasing availability of endovascular technologies, whose design and sizing were largely based on Western anatomical norms and may not optimally accommodate populations with smaller or differently proportioned vascular anatomy. Against this background, a growing body of contemporary literature has begun to explore racial differences in aortoiliac diameters, aneurysm prevalence, and rupture characteristics. However, these data remain fragmented and heterogeneous, and no comprehensive synthesis has yet translated these findings into practical implications for clinical decision-making.

Therefore, this narrative review aims to provide a comprehensive examination of racial and ethnic differences in aortoiliac arterial dimensions and to assess their clinical relevance in the diagnosis of aneurysms and the evaluation of rupture risk. It further critically appraises the limitations of applying uniform diameter thresholds across diverse populations and explores the rationale for population-specific or anthropometrically indexed criteria for defining aneurysmal disease. Finally, this review underscores the pressing need for more inclusive, evidence-based approaches to aneurysm surveillance and intervention.

## 2. Population Variability in Aorto-Iliac Arterial Diameters

Growing evidence indicates that normal aorto-iliac arterial diameters exhibit substantial anatomical variability across racial and ethnic populations. This variability has important implications for the interpretation of vascular dilation and the applicability of universal thresholds used to define aneurysmal disease. Since aneurysms are fundamentally diagnosed based on absolute vessel diameter, even modest differences in baseline arterial size may result in clinically meaningful variations in how aneurysmal pathology is identified, risk-stratified, and managed among diverse populations.

### 2.1. Racial and Ethnic Differences in Aortic Diameter

Normal abdominal aortic diameter demonstrates consistent and reproducible variation across racial and ethnic groups. Importantly, these differences persist even after adjustment for key anthropometric variables such as height, age, and BSA, suggesting that population-specific anatomical patterns reflect not only body habitus but also potential genetic and developmental determinants. Caucasian populations generally exhibit larger infrarenal aortic diameters when compared with East Asian cohorts. Reported mean infrarenal diameters in Caucasian males typically range from approximately 20.5 to 21.4 mm, whereas East Asian males show smaller average diameters of approximately 18.0 to 19.2 mm [11,12,15]. Similar trends are observed in female cohorts, with consistently smaller diameters reported in Korean, Chinese, and Japanese populations relative to Western counterparts [11,12,16,17]. African American and Hispanic populations display distinct baseline dimensions as well, although available data are more limited and heterogeneous [13,14]. The Multi-Ethnic Study of Atherosclerosis (MESA) highlighted that vascular remodeling patterns differ not only between Asians and Caucasians, but also among African American and Hispanic populations, emphasizing the complexity of race-associated anatomical variation [18].

The Framingham Heart Study remains a cornerstone in establishing normative aortic dimensions for Western populations through CT-based measurements, offering widely cited reference values frequently used in guideline formation [19]. In contrast, several Korean studies have consistently demonstrated significantly smaller aortic diameters compared to Western standards, even after indexing to BSA or height [11,12,20]. These findings challenge the assumption that global application of Western-derived thresholds is appropriate across all populations and underscore the potential for systematic misclassification of aneurysmal disease when racial context is not considered.

### 2.2. Updated Evidence

Building upon previously published literature, including a systematic review from 2020 [21], we conducted a structured literature search to capture more recently published studies reporting normal infrarenal aortic diameters. This focused update was undertaken to incorporate contemporary population-level data while preserving the narrative scope of the review. The search was performed in PubMed (MEDLINE) for articles published from January 2020 onward, using combinations of terms related to abdominal aortic diameter, reference values, population, ethnicity, and imaging modality. Studies were considered if they included more than 100 adult participants, reported absolute infrarenal aortic diameters, and used clearly defined ultrasound or computed tomography measurement techniques. The purpose of this structured search was to update key reference data and to illustrate recent geographic and racial trends in aorto-iliac anatomy, rather than to provide an exhaustive systematic synthesis. The characteristics of the studies summarized in this section are presented in Table 1, encompassing diverse populations across Europe, Asia, Africa, Oceania, and North America.

**Figure 1:**
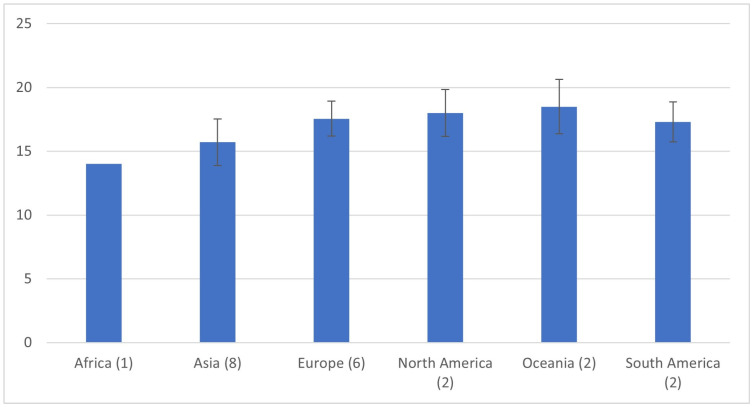
Mean infrarenal aortic diameter by continent, based on pooled data regardless of sex. The number of studies contributing to each continental estimate is indicated in parentheses. Error bars represent standard deviations.

**Figure 2:**
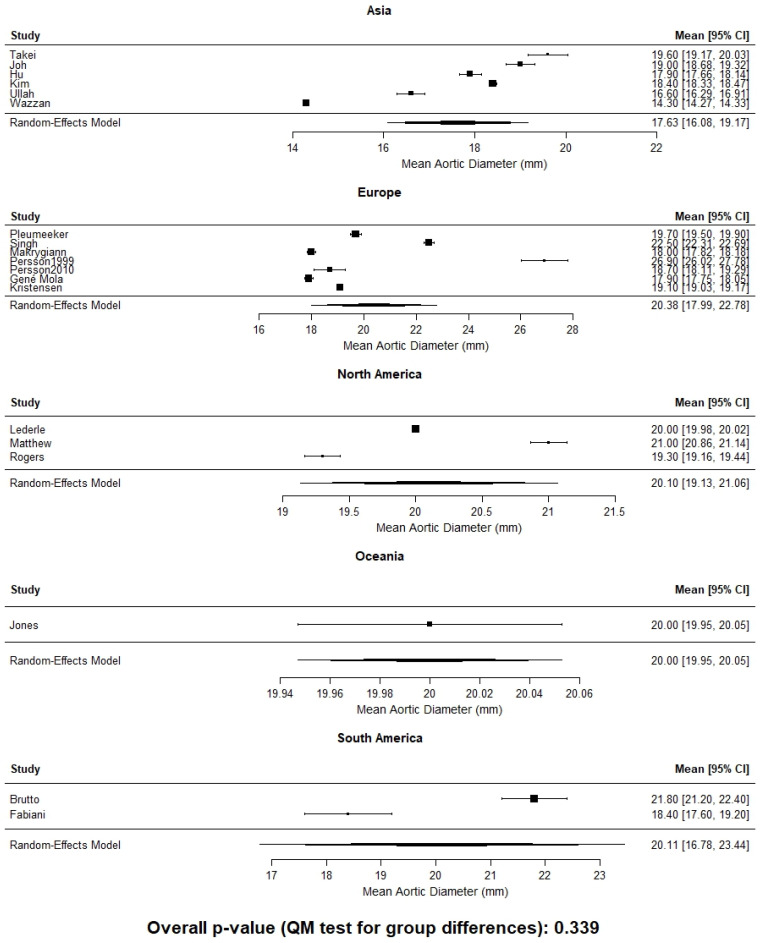
Mean infrarenal aortic diameter by continent in males. No statistically significant differences were observed in continent-level comparisons (*p* = 0.339).

**Figure 3:**
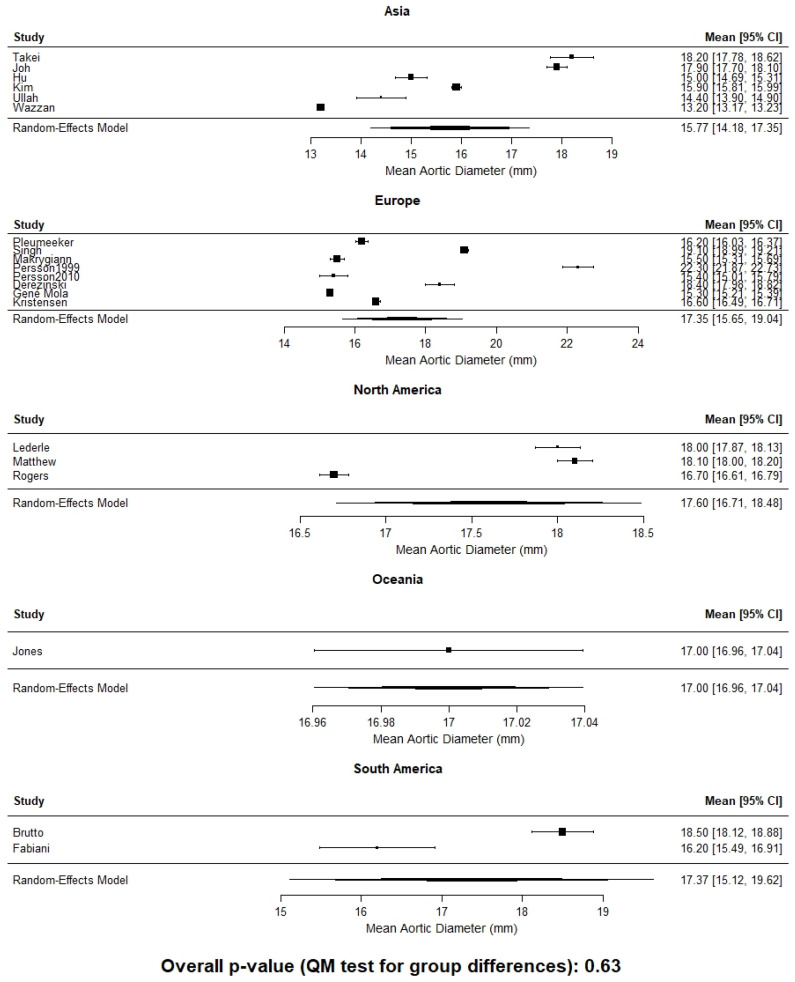
Mean infrarenal aortic diameter by continent in females. No statistically significant differences were observed in continent-level comparisons (*p* = 0.63).

When infrarenal aortic diameter data were pooled without stratification by sex, mean values varied markedly by continent, ranging from approximately 14.0 mm in African cohorts to 18.5 mm in Oceania (Figure 1). This finding likely reflects a combination of genetic background, environmental exposure, nutritional patterns, and methodological differences among studies. Although continent-level comparisons did not demonstrate statistically significant differences when stratified by sex, a consistent trend toward smaller aortic diameters was observed in Asian populations for both males and females (Figure 2 and Figure 3). This finding should be interpreted in the context of substantial heterogeneity within continents and the limited power of ecological comparisons, rather than as evidence against clinically meaningful population-specific differences.

### 2.3. Iliac Artery Diameter Variability

Racial and ethnic variations are also evident in the iliac arteries, which represent distal extensions of the abdominal aorta and play a central role in aneurysmal disease progression and EVAR planning. Reference diameters of the common iliac arteries vary substantially according to population and sex, and these differences become increasingly relevant with the growing recognition and detection of isolated iliac artery aneurysms in aging societies.

Multiple studies have demonstrated that East Asian individuals tend to have significantly smaller common iliac artery diameters compared with Western counterparts. Mean iliac artery diameters in Asian populations are approximately 10.4–12.4 mm in men, compared with 14–15 mm typically reported in Caucasian males, while Korean female cohorts often demonstrate diameters closer to 10 mm, markedly smaller than reference values in Western literature [11,12,15,17,30] (Table 2). Notably, CT-based data from the Framingham Heart Study demonstrate substantially larger common iliac artery diameters in Western populations compared with East Asian cohorts, underscoring the limitations of applying uniform iliac diameter thresholds in EVAR planning across diverse populations. These discrepancies have important clinical implications when applying aneurysm definitions that rely on absolute thresholds, such as ≥18 mm in men and ≥15 mm in women [10]. In smaller-bodied populations, such thresholds may correspond to a much more advanced degree of proportional dilation, potentially delaying intervention relative to pathophysiologic risk. Conversely, in populations with inherently larger iliac diameters, strict adherence to these thresholds may lead to overtreatment.

Although isolated iliac artery aneurysms are less prevalent than abdominal aortic aneurysms, they are more frequently symptomatic and may carry a substantial risk of rupture, often occurring at smaller diameters and associated with high mortality when rupture occurs [31]. Current repair thresholds, typically set at 3.0–3.5 cm, are derived almost exclusively from Western cohorts, and the appropriateness of these criteria in non-Western populations remains inadequately validated [32,33].

These observations highlight the urgent need to define normal and pathological iliac artery diameters using race- and sex-specific normative references. Given the close anatomical and biomechanical relationship between the abdominal aorta and iliac arteries, a combined evaluation framework incorporating both vessel segments may offer superior risk stratification, especially in populations with smaller baseline vascular dimensions. Furthermore, the inclusion of iliac artery data in large population-based imaging studies and international registries would significantly enhance the precision of surveillance strategies, procedural planning, and timing of intervention, ultimately facilitating more equitable and individualized vascular care. Compared with the abdominal aorta, population-based reference data for common iliac artery diameters remain relatively sparse and methodologically heterogeneous. Many studies report iliac measurements only as secondary outcomes, often without standardized anatomical landmarks or consistent sex stratification. As a result, robust continent-level or race-specific comparisons analogous to those performed for the aorta are currently limited. Nevertheless, available data consistently demonstrate smaller iliac artery diameters in East Asian populations compared with Western cohorts, a finding with direct implications for EVAR device selection, limb sizing, and long-term durability.

## 3. Limitations of Fixed Diameter–Based Paradigms

Current clinical guidelines for aneurysm repair recommend intervention at fixed absolute diameter thresholds, most commonly 5.5 cm for the infrarenal abdominal aorta in asymptomatic patients, based largely on randomized controlled trials and observational data derived from Western populations [6,10]. While these thresholds have substantially reduced rupture-related mortality at a population level, their universal applicability across diverse racial, ethnic, and anthropometric groups has become increasingly questioned. A fundamental limitation of this approach lies in the assumption that aneurysm rupture risk is primarily determined by absolute diameter alone. In reality, rupture represents a complex biomechanical event influenced by multiple factors, including wall stress, vessel compliance, intraluminal thrombus, local geometry, growth rate, and baseline vessel dimensions. Populations with smaller normal aortic diameters, such as East Asians, may reach a pathophysiologically critical proportional dilation at diameters well below 5.5 cm. Consequently, aneurysms deemed “moderate” by Western standards may already represent advanced disease in these groups. Several studies suggest that East Asian patients, characterized by smaller baseline aortic sizes, may experience rupture or dissection at dimensions traditionally considered low risk in Caucasian populations [11,34]. This raises concern that strict adherence to Western-derived thresholds could result in delayed intervention and potential under-treatment, exposing certain populations to avoidable rupture risk. Conversely, individuals with larger baseline vascular dimensions, such as taller Caucasian or African American patients, may undergo unnecessary intervention if absolute diameter thresholds are applied without accounting for body habitus or relative aortic enlargement. This one-size-fits-all paradigm does not consider proportional dilation, defined as the ratio between aneurysmal diameter and expected normal vessel size. Several authors have proposed indexing aneurysm size to BSA, height, or predicted normal diameter as a more physiologically relevant marker of rupture risk. Metrics such as the aortic size index (ASI) or percentage increase from baseline diameter may offer improved risk stratification compared with absolute diameter alone, particularly in populations with extreme anthropometric profiles.

Additionally, reliance on fixed thresholds neglects other important predictors of aneurysm instability, including growth rate, aneurysm morphology, eccentricity, and wall composition. Evidence indicates that rapidly expanding aneurysms and those with complex morphologic features may rupture at smaller diameters, further undermining confidence in diameter-based decision-making as the sole determinant of intervention timing. Emerging data therefore support a shift toward individualized thresholds that integrate patient-specific anatomy, demographic background, and biomechanical factors. This transition aligns with broader movements in vascular surgery and cardiovascular medicine toward precision-based care, where treatment decisions are guided not only by population averages but by individualized risk modeling.

Ultimately, the continued application of uniform diameter thresholds across heterogeneous populations may perpetuate inequities in care, particularly for populations that systematically diverge from the anatomical norms upon which these thresholds were originally established. As such, re-evaluation of current criteria through population-specific data and global registries represents a critical step toward safer and more equitable aneurysm management.

## 4. Individualized Threshold Approaches

In response to the growing recognition that universal diameter thresholds inadequately reflect inter-population variation, several authors have proposed alternative approaches that redefine aneurysmal disease based on relative enlargement rather than absolute vessel size [35,36]. This proportional framework aligns with classical vascular definitions and offers a more biologically rational method of identifying pathological dilation across populations with differing baseline anatomy [37,38,39].

Height-indexed and BSA–indexed thresholds have also been suggested as complementary or alternative tools for risk stratification [37,40,41]. Several studies have demonstrated that size indices adjusted for anthropometric parameters correlate more closely with rupture risk than absolute aneurysm diameter alone. Metrics such as the aortic size index (ASI), which normalizes aneurysm diameter to BSA, have shown improved predictive value in identifying high-risk aneurysms, particularly in individuals at the extremes of body habitus. For example, Davies et al. found that patients with an ASI (aortic diameter divided by BSA) > 4.25 cm/m^2^ had markedly higher rupture risk compared to those with ASI < 2.75 cm/m^2^ [40]. More recently, Olukorode et al. confirmed that ASI predicted rupture more accurately than diameter in a large contemporary series [37]. These approaches acknowledge that a 5.5 cm aneurysm may not carry equivalent biomechanical significance in individuals of markedly different stature or baseline vessel size.

Beyond anthropometric indexing, mathematical modeling of arterial geometry and biomechanical stress distribution has further highlighted the limitations of diameter-only evaluation [42]. Finite element analyses and computational modeling techniques suggest that wall stress patterns—influenced by vessel curvature, aneurysm asymmetry, intraluminal thrombus distribution, and wall composition—contribute significantly to rupture risk. Integrating these parameters with individualized anatomical thresholds may allow for more nuanced risk estimation and clinical decision-making.

Z-score assessment, a statistical method that expresses vessel diameter as deviations from population-specific mean values, has been widely adopted in the evaluation of thoracic aortic dimensions, particularly in pediatric populations and patients with connective tissue disorders such as Marfan syndrome or Loeys–Dietz syndrome [43,44,45]. Despite its proven utility in these settings, application of Z-score methodology to abdominal aortic aneurysms remains largely unexplored within current guidelines. Given the documented racial and ethnic variability in baseline infrarenal aortic diameter, extending Z-score principles to the abdominal aorta could offer a more precise and equitable framework for defining abnormal dilation.

Such an approach would allow clinicians to contextualize aneurysm size relative to expected population norms, rather than relying solely on fixed numerical thresholds derived from Western cohorts. This model is particularly appealing for regions with smaller baseline vessel diameters, such as East Asia, where conventional cut-offs may underestimate true aneurysmal burden. Conversely, in populations with larger baseline vascular dimensions, Z-score–based evaluation could prevent premature or unnecessary intervention.

Taken together, these individualized threshold strategies represent an important paradigm shift from population-based averages toward personalized vascular risk assessment. While further validation is required before universal adoption, the integration of relative enlargement criteria, anthropometric indexing, and population-specific Z-score frameworks may form the foundation for future guideline refinement. Such strategies have the potential to improve clinical precision, reduce inequities in care, and align aneurysm management more closely with true pathophysiological risk.

## 5. Clinical and Research Implications

While the evidence for sex-based differences in aneurysm rupture risk is well-established, it also serves as a powerful analogy in support of adopting race-specific thresholds. Current guidelines, such as those by the SVS and ESVS, now recommend lower intervention thresholds for women, acknowledging their smaller baseline aortic diameters and higher rupture risk at any given absolute diameter [6,10]. This shift reflects an understanding that fixed diameter thresholds do not account for anatomical variability. Using the same logic that justifies lower thresholds in women, it is reasonable to advocate for race-adjusted or indexed thresholds that reflect the baseline vessel size and risk profile of each population. These refinements could improve the precision of aneurysm management and align treatment decisions with individualized patient risk, as current guidelines are beginning to do for sex-based differences. Moreover, several additional lines of evidence further support the need for race-adjusted thresholds:

First, aneurysms are fundamentally defined as abnormal dilations relative to the individual’s baseline vessel diameter, commonly accepted as a ≥50% increase from normal [6,10]. Second, population-specific studies from East Asia have shown that rupture can occur at sizes below 5.5 cm, suggesting that the current thresholds derived from predominantly Caucasian cohorts may not be universally safe [46]. Third, indexing to height or BSA may provide more equitable thresholds. These indices are already shown to correlate more closely with rupture risk across both sexes and diverse body sizes, and may similarly benefit racial and ethnic risk stratification [39,47]. Finally, the foundational trials that established current thresholds and reported no significant survival benefit from early elective repair for AAAs between 4.0 and 5.5 cm [7,48]. However, these trials assumed high compliance with rigorous ultrasound surveillance (every 3–6 months), which may not be feasible in many real-world or resource-limited settings. Additionally, these studies did not consider racial differences in baseline aortic size or EVAR eligibility over time. Their findings, while robust, may not generalize to other groups without adjustment or further validation.

Further supporting this need, a series of more recent trials such as CAESAR and PIVOTAL reaffirmed the safety of surveillance in well-controlled Western populations with small AAAs. Yet, subgroup analyses show that most patients with AAAs measuring 5.0–5.5 cm eventually undergo repair within 3–4 years due to aneurysm growth [49,50]. Moreover, up to 16% may lose eligibility for standard EVAR due to progressive anatomical changes such as neck angulation or shortening These findings suggest that delayed intervention may lead to missed opportunities for simpler, safer procedures, especially in patients with initially borderline EVAR anatomy.

Rupture of AAAs smaller than guideline thresholds is also not uncommon. Recent registry data show that approximately 10% of ruptured AAAs occur in males with <5.5 cm aneurysms and in females with <5.0 cm [51]. Notably, these patients tend to be younger, non-white, and have more comorbidities such as diabetes and renal failure—suggesting racial and socioeconomic disparities in disease monitoring and access to care. Collectively, these data highlight the need to reconsider a single absolute aneurysm threshold and to move toward population-appropriate, individualized criteria.

While the rationale for population-specific aneurysm thresholds is increasingly supported by anatomical and epidemiological data, their clinical implementation must be both evidence-based and practical. Rather than introducing fixed race-specific diameter cutoffs, a more feasible approach may involve incremental integration of proportional and indexed metrics into existing decision frameworks.

One pragmatic strategy is the incorporation of anthropometrically indexed measures, such as height- or body surface area–adjusted diameters and the aortic size index, which can be readily calculated using routinely available clinical data. These metrics allow aneurysm size to be interpreted relative to expected baseline vessel dimensions, reducing bias introduced by inter-population differences in body habitus.

A complementary approach involves the development of population-specific Z-score reference ranges derived from large, multiethnic imaging registries. Z-score methodology is already well established in thoracic aortic disease and could be extended to the abdominal aorta and iliac arteries, enabling standardized interpretation of vessel enlargement relative to normative datasets.

Finally, implementation may occur through stepwise guideline refinement, in which indexed or relative enlargement criteria are first introduced as adjunctive risk modifiers rather than replacement thresholds. Such a framework would preserve the safety of existing guidelines while allowing gradual transition toward more individualized, population-appropriate aneurysm management.

## 6. Discussion

Racial and ethnic variability in aorto-iliac diameters represents an increasingly important topic in vascular medicine, particularly as endovascular therapy becomes the dominant modality for aneurysm repair worldwide.

A major limitation of the current literature is the heterogeneity of study design, imaging modality, and patient selection. Most studies are retrospective, single-center, or rely on imaging obtained for unrelated clinical indications, leading to potential selection bias [52]. Furthermore, computed tomography—while highly accurate—is not uniformly standardized across institutions, and differences in contrast phases and reconstruction algorithms may introduce additional variability.

A more explicit link between baseline vessel diameter and rupture risk further supports the clinical relevance of population-specific reference values. Aneurysm rupture is influenced not only by absolute diameter but also by the degree of proportional enlargement relative to baseline vessel size, which determines wall stress distribution. In populations with smaller native aortic diameters, such as East Asian cohorts, a given absolute aneurysm size represents a greater relative dilation and may therefore reach biomechanical instability at smaller diameters. This concept is supported by observational studies reporting rupture or symptomatic presentation at diameters below conventional thresholds, particularly in populations with smaller baseline aortas and in women, who similarly exhibit higher rupture risk at smaller absolute diameters. Although robust race-specific rupture data remain limited, these findings suggest that baseline anatomical differences modify the relationship between aneurysm size and rupture risk, reinforcing the limitations of uniform diameter thresholds and supporting the rationale for indexed or population-adjusted criteria.

Another important issue is the lack of standardized reference values across age, sex, and body size. Although BSA, height, and overall body habitus differ substantially across racial groups, most published reference ranges do not adjust for these anthropometric variables. As a result, it is unclear how much of the observed difference is attributable to racial or genetic factors versus differences in body composition. Studies evaluating the utility of indexed metrics—such as the aortic size index (ASI)—suggest that such adjustments may better stratify risk, but data remain limited, especially outside Western populations [38,40].

The clinical implications of using a single global threshold are potentially significant. This discrepancy could contribute to differences in rupture risk, device failure, and long-term outcomes, yet robust outcome data comparing racial groups remain scarce.

Emerging data suggest that risk-stratification strategies should consider population-specific baseline diameters, sex-specific thresholds, and possibly indexed measurements. However, the evidence remains insufficient to define race-specific intervention criteria. The absence of large, prospective, multiethnic imaging registries represents a major barrier to resolving these uncertainties.

Lastly, the literature demonstrates a marked geographic imbalance, with limited data from regions such as South Asia, the Middle East, South America, and Africa [53]. The near-absence of population-level reference data from these areas limits generalizability and contributes to uncertainty in global clinical practice.

Overall, the current body of evidence highlights meaningful anatomical differences between racial groups, but gaps in methodology, sample diversity, and outcome correlation underscore the need for more rigorous research to determine how these differences should influence aneurysm diagnosis, surveillance, and intervention thresholds.

### Limitations

This review has several limitations that should be considered when interpreting its findings. First, the manuscript is structured as a narrative review. Although a structured literature search with predefined criteria was applied to selected sections, the review does not claim exhaustive retrieval of all available studies. Consequently, the synthesis reflects representative contemporary evidence rather than a comprehensive systematic assessment.

Second, potential selection bias and geographic imbalance exist within the available literature. Many studies reporting aorto-iliac diameters are retrospective or derived from population-based screening programs and imaging performed for other clinical indications. In addition, certain regions—particularly low- and middle-income countries—remain underrepresented, limiting the generalizability of population-level reference values and reinforcing the need for more globally inclusive imaging registries.

Third, there is methodological heterogeneity in imaging acquisition and measurement practices across studies. While most investigations clearly reported the imaging modality used (ultrasound or computed tomography), detailed CT acquisition parameters and explicit diameter definitions (e.g., inner-to-inner versus outer-to-outer measurements) were not uniformly specified. In practice, particularly in non-aneurysmal or healthy aortas, differentiation between inner and outer wall boundaries on CT is often limited and unlikely to materially influence reported reference diameters. Moreover, none of the included population-based studies systematically reported or adjusted aortic diameters for body size variables such as height or body surface area. Instead, absolute diameters were consistently used, reflecting current reporting standards in large screening and imaging cohorts. These factors contribute to between-study heterogeneity and underscore the need for standardized measurement protocols and indexed reference frameworks in future research.

Finally, analyses relying on continent-level aggregation are inherently subject to ecological fallacy, as continents encompass heterogeneous racial, ethnic, and anthropometric populations. Accordingly, the absence of statistically significant differences in continent-level comparisons should not be interpreted as evidence against clinically meaningful population-specific anatomical variation. Individual-level, multiethnic data are required to more accurately define race- or population-appropriate reference standards.

## 7. Future Direction

Future research addressing racial and ethnic variability in aorto-iliac diameters should prioritize the development of robust, standardized, and globally inclusive evidence. A central need is the establishment of large-scale, prospective, multiethnic imaging registries using harmonized measurement protocols. Such registries would allow valid cross-population comparisons by minimizing the methodological inconsistencies that currently hinder interpretation of vessel size variation, growth patterns, and aneurysm prevalence.

Standardization of imaging and measurement techniques represents another critical priority. Existing studies differ in modality choice and diameter definitions—such as inner-to-inner versus outer-to-outer measurements—resulting in substantial heterogeneity. Adoption of uniform imaging protocols, supported by automated or AI-assisted analytics, would enhance measurement reproducibility and facilitate integration of data across centers and populations.

Clarifying the contributions of anthropometric, genetic, and lifestyle factors to observed diameter differences will also be essential. Incorporating body size indices, genetic markers, and environmental exposures into future study designs may help differentiate intrinsic racial anatomical differences from modifiable or confounding factors. These efforts may also support the development of indexed thresholds, such as BSA-adjusted diameters or Z-score–based assessments, which could provide more precise risk stratification than absolute diameter cutoffs.

Outcome-based research is another key direction. Prospective studies evaluating whether baseline anatomical differences translate into distinct rupture risks, growth trajectories, device performance, or procedural complications are necessary to determine whether current intervention thresholds are appropriate for all racial groups. Evaluating whether earlier diagnosis or lower thresholds improve outcomes in these populations should be a research priority.

Device design must also evolve to reflect global anatomical diversity. Most EVAR systems have been engineered based on Western vessel dimensions, which may not optimally accommodate patients with smaller or differently proportioned aorto-iliac anatomy. Engineering innovations targeting graft sizing, fixation mechanisms, iliac limb dimensions, and delivery system profiles tailored to a broader range of vascular anatomies could improve procedural success and reduce complications, particularly among Asian patients.

Finally, meaningful progress will depend on coordinated, cross-disciplinary, and international collaboration. Partnerships among vascular surgeons, radiologists, epidemiologists, biomechanical engineers, and global health researchers will be essential to implement harmonized research frameworks, validate population-specific thresholds, and ensure that evolving diagnostic and therapeutic strategies equitably address the needs of diverse patient populations.

Taken together, these research priorities underscore the necessity of refining current aneurysm definitions and management pathways to better accommodate global anatomical variability, ultimately improving diagnostic accuracy, procedural safety, and long-term outcomes across racially diverse populations.

## 8. Conclusions

There is increasing recognition that the fixed diameter thresholds currently used to guide aortic and iliac aneurysm repair may not be universally appropriate. Applying uniform size thresholds across diverse populations risks either delaying treatment in high-risk patients or promoting unnecessary interventions in others.

To improve care, more inclusive research is urgently needed to establish race- and sex-specific reference values and to validate alternative intervention criteria indexed to body size or anatomical variation. Thresholds for iliac artery aneurysm diagnosis and repair, in particular, warrant re-evaluation, given the significant size differences observed across ethnic groups. Ultimately, tailoring aneurysm management to reflect global diversity will reduce rupture risk, improve patient outcomes, and enhance cost-effective care.

## Figures and Tables

**Table 1:** Summary of included studies.

Author	Year	Country	Continent	Population	N	Age	Modality	AD (mm)
Derezinski (F) [22]	2020	Poland	Europe	Caucasian women, aged 65–74	200	69.5	US	18.4 ±3
Ezenwugo [23]	2020	Nigeria	Africa	>18 years	400	NR	US	14.0 ± 2
Jones (M) [24]	2020	New Zealand	Oceania	>50 years (AAA screening program)	1118	69.4	US	20.0 (18.3–22.0)
Jones (F) [24]	2020	New Zealand,	Oceania	>50 years (AAA screening program)	1992	69.4	US	17.0 (15.4–18.8)
Gené Mola (M) [25]	2023	Spain	Europe	=65 years AAA screening program	2089	65	US	17.9 ± 3.5
Gené Mola (F) [25]	2023	Spain	Europe	>65 years AAA screening program	2641	65	US	15.3 ± 2.3
Kristensen (M) [26]	2023	Denmark	Europe	Viborg Vascular Screening Trial	19,269	69.6	US	19.1 ± 5.3
Kristensen (F) [26]	2023	Denmark	Europe	Viborg Vascular Screening Trial	2426	67.1	US	16.6 ± 2.8
Spanos (M) [27]	2023	Greece	Europe	Males > 60 years (AAA screening program)	1187	71	US	18 ± 2
Rogers (M) [19]	2013	USA	North America	Framingham Heart Study	1767	49.8	CT	19.3 ± 2.9
Rogers (F) [19]	2013	USA	North America	Framingham Heart Study	1664	52.2	CT	16.7 ± 1.8
Fabiani (M) [13]	2022	Mexico	South America	Retrospective study	51	49.6	CT	18.4 ± 2.9
Fabiani (F) [13]	2022	Mexico	South America	Retrospective study	55	49.6	CT	16.2 ± 2.7
Hu (M) [17]	2022	China	Asia	Retrospective study	380	60	CT	17.9 ± 2.4
Hu (F) [17]	2022	China	Asia	Retrospective study	245	61	CT	15.0 ± 2.5
Kim (M) [12]	2022	Korea	Asia	Retrospective study	2379	56.8	CT	18.4 ± 1.8
Kim (F) [12]	2022	Korea	Asia	Retrospective study	1313	58.1	CT	15.9 ± 1.7
Ullah (M) [28]	2022	Pakistan	Asia	Retrospective study	194	39.5	CT	16.6 ± 2.2
Ullah (F) [28]	2022	Pakistan	Asia	Retrospective study	56	40.2	CT	14.4 ± 1.9
Wazzan (M) [29]	2022	Saudi Arabia	Asia	Retrospective study	160	50.8	CT	14.3 ± 0.2

AD, aortic diameter; US, ultrasound; CT, computed tomography; F, female; M, male. Sex-specific sample sizes are presented in separate rows where available. For studies in which sex distribution was not reported in the published abstract or full text, data are indicated as not reported (NR).

**Table 2:** Reference common iliac artery diameters across different populations.

Author	Year	Country	Population	N	Modality	CIA Diameter (M, mm)	CIA Diameter (F, mm)
Joh [11]	2013	Korea	Healthy adults	1229	US/CT	11.9 ± 1.8	10.3 ± 1.5
Kim [20]	2022	Korea	Healthy cohort	3692	CT	12.4 ± 1.9	10.1 ± 1.6
Hu [17]	2022	China	Middle-aged/elderly	625	CT	11.2 ± 1.7	9.8 ± 1.5
Rogers [19]	2013	USA	Framingham	3431	CT	14.2 ± 2.1	12.7 ± 1.8
Wazzan [29]	2022	Saudi Arabia	Healthy adults	160	CT	12.3 ± 1.9	10.8 ± 1.6

## Data Availability

No new data were created or analyzed in this study. Data sharing is not applicable to this article.

## Abbreviations

The following abbreviations are used in this manuscript:

AAA	Abdominal aortic aneurysm
ACC/AHA	American College of Cardiology/American Heart Association
ASI	Aortic size index
BSA	Body surface area
CT	Computed tomography
ESVS	European Society for Vascular Surgery
EVAR	Endovascular aneurysm repair
SVS	Society for Vascular Surgery
US	Ultrasound
